# Does the implementation of a national oral health policy reduce inequalities in oral health services utilization? The Brazilian experience

**DOI:** 10.1186/s12889-021-10586-2

**Published:** 2021-03-19

**Authors:** Maria Helena Rodrigues Galvão, Angelo Giuseppe Roncalli

**Affiliations:** Department of Dentistry, Federal University of Rio Grande do Norte, Natal, Rio Grande do Norte Brazil

**Keywords:** Healthcare disparities, Oral health, Health policy

## Abstract

**Background:**

This study aimed to assess the trend in income-related inequalities in oral health services utilization by the Brazilian population from 1998 to 2013. This period represents a timeline that includes different stages of implementation of the National Oral Health Policy.

**Methods:**

The design was based on repeated cross-sectional surveys using secondary data from household-based studies carried out in Brazil in 1998, 2003, 2008, and 2013. The dependent variable was “having access to a dentist appointment at least once in a lifetime (yes/no).” Monthly household per capita income, based on Brazil’s minimum wage, was included as the main independent variable. To measure the inequalities in oral health access related to economic position, the following complex indexes based on regression were used: (a) the slope index of inequality (SII) and (b) the relative index of inequality (RII).

**Results:**

There was a reduction in the percentage of individuals who never had a dentist appointment for all age groups and income classifications. In general, there was a reduction trend in absolute inequality for all age groups (*p* < 0.001). The relative inequality and reduction trend were different between the age groups studied.

**Conclusions:**

The National Oral Health Policy was very important for expanding free of charge, public access to dental appointment. However, despite policy implementation, there continues to be high levels of inequality in access to dental consultation. Assessing which strategies are necessary to overcome this challenge is discussed.

## Background

The utilization of dental services is associated with many factors, including socioeconomic characteristics [1, 2]. Inequalities in the use of dental services are seen globally and have resulted in limited access to dental care for billions of people [2]. The main strategy for mitigating these inequalities is the implementation of oral health policies. In general, these policies could improve access to dental services, although it is most challenging to reduce the social and economic gradients in oral health care systems to produce a more egalitarian and just society [3].

In Brazil, health assistance is considered a citizenship right, being offered through a universal public health system guaranteed by the 1988 national constitution. Since 2004, Brazil has also developed a specific oral health policy, namely, the National Oral Health Policy (PNSB, in Portuguese), better known as *Smiling Brazil* (*Brasil Sorridente*, in Portuguese). This policy represents an innovative strategy focused on potentializing oral health care access across the country, among other initiatives. It is based on two different, but complementary strategies: increasing the number of oral health teams in primary care (especially in the *Family Health Strategy*) and expanding the secondary level of oral health care through the Centers of Specialized Dentistry (Centros de Especialidades Odontológicas – CEO, in Portuguese). The main objective of these two approaches is to increase integral health care, offering oral health services at different levels of complexity [4].

Since the implementation of this policy, an expansion in the number of oral health teams in primary care (ESB, from the Portuguese acronym) from 607 teams in 2003 to 25,905 in 2017 was observed, resulting in population coverage of 41.2%. The population coverage is estimated by calculating the proportion of the population covered by ESB in relation to the whole population, considering a parameter of 3450 patients per provider determined by the Ministry of Health [5]. Regarding the expansion of secondary care, 1134 Centers of Specialized Dentistry (CEO) were established between 2004 and 2019, significantly increasing the availability of oral health services for the Brazilian population [6].

In relation to the oral health epidemiological profile, there was a decline in both prevalence and severity of dental caries, yet inequalities in distribution of these variables persisted in Brazil. This phenomenon was described by Antunes et al. [7] as “polarization of the caries experience.” For adolescents, Roncalli et al. [8] observed that the significant reduction in dental caries observed between 2003 and 2010 was followed by an increase in inequality, meaning that there was a higher reduction in dental caries for the wealthy compared to the poor.

Regarding oral health care access, the last Brazilian National Health Survey (PNS, from the Portuguese acronym), conducted in 2013, showed that 112 million Brazilians (55.6%) did not have access to oral health services in the 12 months prior to the interview. This percentage rose to 61.8% for Black people and to 63.4% for those without a formal education [9].

Despite the implementation of an oral health public policy as part of a universal health system, there is still a lack of knowledge about oral health care utilization by the Brazilian population, especially regarding socioeconomic inequalities.

Thus, this study aimed to assess the trend in income-related inequalities in oral health services utilization by the Brazilian population from 1998 to 2013. This period represents a timeline that includes the following stages in the implementation the National Oral Health Policy: 5 years before implementation (1998), the start of implementation (2003), and 5 years (2008) and 10 years (2013) after the first initiatives of *Smiling Brazil*. Although it seems impossible to attribute changes in inequality trends uniquely to the implementation of the National Oral Health Policy, this type of analysis is fundamental to the establishment of strategies focused on a more equitable oral health care system.

## Methods

The study was a secondary data analysis of responses from household-based repeated cross-sectional surveys carried out in Brazil. Data from the health supplement of the National Household Sample Survey (Pesquisa Nacional Por Amostra de Domicílios – PNAD, in Portuguese) of 1998, 2003, and 2008, and from the National Health Survey (Pesquisa Nacional de Saúde – PNS, in Portuguese) of 2013 were included. These data have national comprehensiveness and are representative of the Brazilian population. The four databases that were used consisted of a total sample of 1,327,223 participants. This study was conducted with information available to the public domain, through a dataset, thus ethical approval was not required. More details about the methods and data sources have been published elsewhere [9–12].

The datasets supporting the conclusions of this article are available on the website of the Brazilian Institute of Geography and Statistics (IBGE, in Portuguese). Table 1 presents the main characteristics of the household-based cross-sectional surveys.

**Table 1:** Methodological characteristics of the surveys

	National Household Sample Surveys (PNAD)			National Health Survey (PNS)
	1998	2003	2008	2013
Number of individuals	344,975	384,834	391,868	205,546
Number of households	112,434	133,255	150,591	81,767 selected 62,986 interviewed
Number of cities	793	851	851	6069 Primary Sample Units (PSU)
Data collection period	September 1998	September 2003	September 2008	July 2013
Sampling method	Random sampling of conglomerates	Random sampling of conglomerates	Random sampling of conglomerates	Random sampling of conglomerates

Source: Instituto Brasileiro de Geografia e Estatística

The dependent variable selected was the answer to the question, “When did you last have a dentist appointment?” The response options were “less than 1 year,” “1 year to 2 years,” “3 or more years,” or “never.” These responses were then categorized as “Having access to a dentist appointment at least once in a lifetime (yes/no).” This outcome was selected due to the fact that it is an important question related to the use of dental services. Taking into account the recommendations for dental assistance [13, 14], responses to this question could reflect the absence of access.

Monthly household per capita income, based on Brazil’s minimum wage, was used as the main independent variable. To create this variable, the original one named “amount of monthly household income” was divided by the number of residents. The original value of Brazilian currency (Reais) was converted into minimum wage, considering the value from the respective year. Finally, the income variable was categorized into five classifications: (1) up to 1.00 minimum wage; (2) 1.00 to 1.99 minimum wages; (3) 2.00 to 2.99 minimum wages; (4) 3.00 to 3.99 minimum wages; and (5) 4 minimum wages or more. Minimum wage was used as a criterion for income standardization because it is a national parameter and represents a social right guaranteed by the Brazilian national constitution. Its value is annually established by legal rules and should be able to supply the basic needs for life. The minimum wage in the selected survey years corresponded to 113 USD in 1998, 84 USD in 2003, 253 USD in 2008, and 317 USD in 2013, considering the currency variation in relation to the US dollar.

The analysis was stratified by age because this variable has an important influence on access to oral health services. Thus, four strata representing the different stages of the life course were created based on the World Health Organization groups: (a) children (0 to 9 years old), (b) adolescents (10 to 19 years old), (c) adults (20 to 59 years old), and (d) elderly (60 years and older).

The four surveys used a complex sample design with clustering defined by the primary sample unit (PSU) and a weight for each participant defined by the selection probability. To calculate the main estimates, the “survey data analysis” module of the software Stata 14 (*svy* command) was used, including the PSU and subject weight in the survey design for the dataset. Descriptive statistics were calculated, such as percentage frequencies and their respective 95% confidence intervals, as well as the expansion of these estimates to the whole Brazilian population.

For measuring the inequalities in oral health access related to economic position, the following complex indexes for the assessment of health inequalities based on regression were used: (a) the slope index of inequality (SII), which estimates the absolute difference between the values of a health indicator in the most privileged and in the least privileged groups, taking into account the other groups, using a regression model, and (b) the relative index of inequality (RII), which estimates the ratio between the estimated values of a health indicator in the most privileged and in the least privileged groups, taking into account the other groups, using a linear regression model in this case, the exponentiated coefficient [15].

To illustrate the main results, *Equiplots* were drawn to demonstrate health inequalities in a given year. The *Equiplot* is a graphic representation of the trend in absolute inequality between groups, developed by the International Center for Equity in Health [16].

## Results

**Fig. 1:**
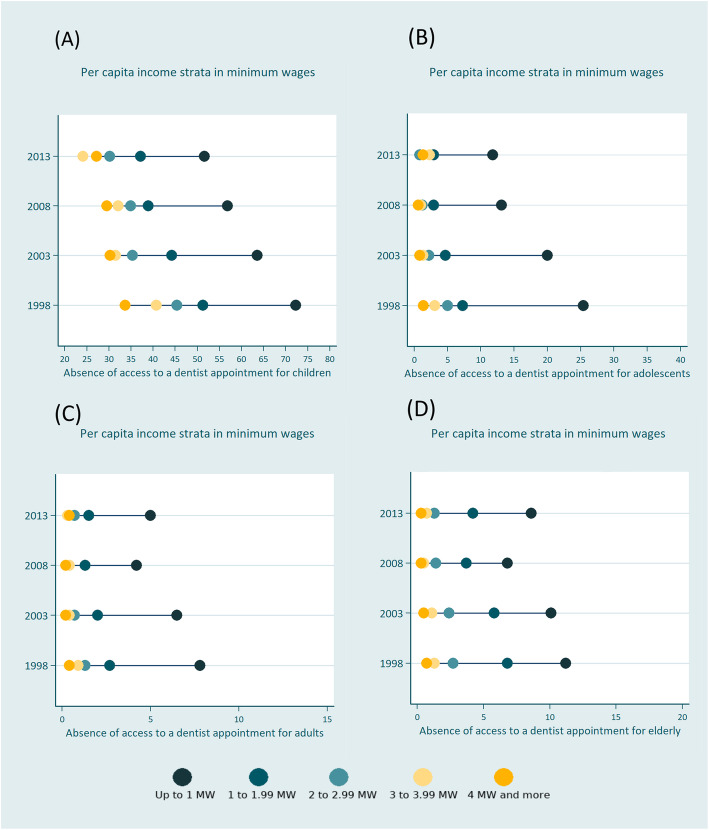
*Equiplots* for not having access to a dentist appointment at least once in a lifetime according to per capita income strata in Brazil from 1998 to 2013. **a** Children; **b** Adolescents; **c** Adults; **d** Elderly

Table 2 shows the population estimates for the lack of access to dentist appointments for the four age groups in the four survey years (1998, 2003, 2008, and 2013). In general, there was a reduction in the percentage of individuals who never had a dentist appointment for all groups and income classifications (Fig. 1).

**Table 2:** National estimates for never having access to a dentist appointment according to age group, per capita income, and year of study

Age group	Household per capita income	Individuals that never had access to a dentist appointment							
		1998		2003		2008		2013	
		n	% (95%CI)	N	% (95%CI)	n	% (95%CI)	n	% (95%CI)
Up to 9 years-old	Up to 1 MW	32,193	72.3 (70.7–73.8)	34,436	63.6 (62.4–64.7)	27,087	56.8 (55.7–57.8)	12,018	51.6 (50.1–53.0)
	1 to 1.99 MW	5849	51.3 (49.9–52.6)	3879	44.2 (42.9–45.5)	3117	38.9 (37.5–40.2)	1644	37.2 (34.9–39.7)
	2 to 2.99 MW	1606	45.4 (42.4–48.4)	937	35.3 (33.0–37.6)	765	34.9 (32.7–37.2)	413	30.2 (26.3–34.4)
	3 to 3.99 MW	761	40.7 (38.1–43.3)	350	31.5 (28.5–34.7)	341	32.1 (29.2–35.3)	144	24.1 (18.9–30.3)
	4 MW and more	1133	33.7 (31.1–36.4)	626	30.3 (28.0–32.7)	454	29.5 (26.8–32.3)	253	27.2 (22.8–32.2)
	Total	41,542	64.0 (62.2–65.8)	40,228	58.1 (57.0–59.2)	31,764	52.3 (51.3–53.2)	14,472	46.8 (45.6–48.1)
10–19 years old	Up to 1 MW	8451	25.4 (23.7–27.3)	10,103	20.0 (18.7–21.2)	6570	13.1 (12.2–14.0)	3425	11.8 (10.9–12.9)
	1 to 1.99 MW	875	7.3 (6.6–8.0)	600	4.7 (4.2–5.2)	385	2.9 (2.6–3.3)	245	2.9 (2.2–3.7)
	2 to 2.99 MW	177	5.0 (4.1–6.0)	89	2.2 (1.7–2.8)	45	1.2 (0.9–2.2)	24	0.8 (0.4–1.4)
	3 to 3.99 MW	66	3.1 (2.4–3.9)	26	1.3 (0.8–2.0)	12	1.0 (0.5–2.0)	5	2.3 (0.5–9.5)
	4 MW and more	67	1.4 (1.0–1.8)	25	0.8 (0.5–1.2)	14	0.6 (0.4–1.1)	9	1.3 (0.5–3.4)
	Total	9636	17.1 (15.5–18.8)	10,843	14.8 (13.8–15.9)	7026	9.9 (9.2–10.7)	3708	8.8 (8.1–9.6)
20–49 years old	Up to 1 MW	5365	7.8 (6.9–8.7)	6635	6.5 (6.0–7.1)	4595	4.2 (3.9–4.5)	3071	5.0 (4.6–5.6)
	1 to 1.99 MW	1153	2.7 (2.4–3.1)	969	2.0 (1.8–2.2)	762	1.3 (1.2–1.5)	579	1.5 (1.3–1.8)
	2 to 2.99 MW	228	1.3 (1.2–1.5)	122	0.7 (0.6–0.9)	68	0.4 (0.3–0.5)	85	0.7 (0.5–1.0)
	3 to 3.99 MW	90	0.9 (0.7–1.3)	30	0.4 (0.2–0.6)	30	0.4 (0.3–0.6)	25	0.3 (0.1–0.5)
	4 MW and more	87	0.4 (0.3–0.6)	42	0.2 (0.2–0.3)	29	0.2 (0.1–0.3)	20	0.4 (0.2–0.9)
	Total	6923	4.4 (3.8–5.0)	7798	4.0 (3.6–4.4)	5484	2.6 (2.4–2.8)	3780	3.0 (2.8–3.3)
60 years and older	Up to 1 MW	936	11.2 (10.0–12.5)	1115	10.1 (9.0–11.4)	863	6.8 (6.2–7.5)	695	8.6 (7.2–10.1)
	1 to 1.99 MW	639	6.8 (5.9–7.8)	747	5.8 (5.1–6.5)	631	3.7 (3.4–4.1)	447	4.2 (3.5–5.0)
	2 to 2.99 MW	84	2.7 (2.1–3.4)	82	2.4 (1.9–3.1)	63	1.4 (1.0–1.9)	49	1.3 (0.9–2.1)
	3 to 3.99 MW	22	1.3 (0.7–2.3)	21	1.1 (0.7–1.9)	14	0.5 (0.3–0.9)	13	0.7 (0.2–2.3)
	4 MW and more	27	0.7 (0.5–1.1)	19	0.5 (0.3–0.9)	16	0.3 (0.2–0.6)	13	0.3 (0.1–0.8)
	Total	1708	6.1	1984	6.0 (5.3–6.6)	1587	3.9 (3.6–4.2)	1217	4.8 (4.2–5.5)
Total	Up to 1 MW	48,705	29.3 (28.2–30.5)	52,289	23.5 (22.7–24.3)	39,115	17.6 (17.1–18.2)	19,209	12.8 (12.2–13.4)
	1 to 1.99 MW	8799	11.1 (10.7–11.6)	6195	7.5 (7.3–7.8)	4895	5.3 (5.1–5.5)	2915	4.2 (3.9–4.5)
	2 to 2.99 MW	2163	7.4 (7.0–7.9)	1230	4.4 (4.1–4.7)	941	3.2 (3.0–3.5)	571	2.3 (2.0–2.6)
	3 to 3.99 MW	955	6.0 (5.5–6.5)	427	3.2 (2.8–3.5)	397	3.0 (2.7–3.3)	187	1.7 (1.3–2.2)
	4 MW and more	1331	3.7 (3.5–4.0)	712	2.7 (2.5–3.0)	513	2.2 (2.0–2.4)	295	1.7 (1.4–2.1)
	Total	61,953	18.9 (17.6–20.3)	60,853	16.0 (15.4–16.7)	45,681	11.9 (11.4–12.3)	23,177	8.2 (7.9–8.6)

*CI* Confidence interval, *MW* Minimum wage

Analyzing the inequality in access to dental services (Table 3), there was a reduction trend in absolute inequality for children. The SII was 54.2 in 1998 and 35.1 in 2013. The relative inequality in children, however, remained constant with an RII of 2.66 in 1998 and 2.31 in 2013. In adolescents, adults, and the elderly, there was a decreasing trend in absolute inequality between 1998 and 2008 with an increase in 2013. The RII for adolescents and adults showed an upward trend in relative inequality in 2003, with a decrease in subsequent years.

**Table 3:** Slope Index of Inequality (SII) and Relative Index of Inequality (RII) for never having access to a dentist appointment

Age group	1998	2003	2008	2013	*P*-value
Slope Index of Inequality (95% confidence interval (CI))					
Up to 9 years old	54.20 (51.93–56.56)	50.78 (48.46–53.10)	39.94 (37.58–42.31)	35.06 (31.88–38.22)	< 0.001
10–19 years old	31.60 (30.92–32.28)	17.67 (17.02–18.31)	18.20 (17.72–18.67)	19.17^a^ (18.58–19.75)	< 0.001
20–49 years old	9.41 (9.16–9.67)	9.06^a^ (8.86–9.26)	5.93 (5.77–6.09)	7.17^a^ (6.12–7.44)	< 0.001
60 years and older	15.80 (14.83–16.78)	14.83 (13.82–15.85)	10.18 (9.42–10.93)	12.35 (11.23–13.46)	< 0.001
Relative Index of Inequality (95%CI)					
Up to 9 years old	2.66 (2.53–2.79)	2.80 (2.65–2.97)	2.42 (2.28–2.57)	2.31 (2.11–2.52)	< 0.001
10–19 years old	27.06 (24.06–30.44)	41.77 (35.50–49.16)	40.10 (31.61–49.30)	26.19 (20.08–34.16)	< 0.001
20–49 years old	24.07 (21.33–27.16)	30.81 (26.97–35.20)	27.17 (23.27–31.74)	18.40 (15.49–21.85)	< 0.001
60 years and older	11.59 (9.49–14.16)	8.60 (7.16–10.34)	9.35 (7.61–11.49)	8.48 (6.71–10.70)	< 0.001

The “*p*” value indicates the significance for the time trend

^a^Convergence not achieved

## Discussion

The findings from the present study demonstrate that there was a reduction in the percentage of individuals who have never had a dentist appointment in Brazil during the survey periods. There was also a tendency toward reduction of absolute inequality, especially among children and adolescents, while there was a lower reduction in relative inequality with differences in these trends between the age groups. Despite this, the importance of income for access to oral health services was observed (i.e., the lower the income, the lower the frequency of having at least one dentist appointment in one’s lifetime), regardless of the year assessed.

Scientific evidence for health inequalities is important to subsidize the process of planning and implementation of public health policies, which must include strategies to reduce the effect of social gradient on the distribution of health conditions [17]. Inequalities in oral health care utilization in Brazil were also revealed in a study conducted by Celeste et al. [18] that used data from oral health epidemiological surveys in Brazil in 1986 and 2003. A reduction in both absolute and relative inequality over time was verified for the 35–44 year old age group. A higher frequency of access for the wealthiest group was found and this effect persisted even after adjusting for confounding factors, such as gender and oral health condition measured by the decayed, missing, and filled teeth (DMF-T) index. Other oral health outcomes, such as self-perception of oral treatment needs, have been analyzed [19] and, in general, such an outcome has significant social determinants at both the individual and contextual levels [20].

Contextual factors have also been associated with the utilization of oral health services in Brazil. Vieira et al. [21] applied multilevel modeling to study this association among adults living in capital cities, using data from the National Oral Health Survey (SBBrasil Project 2010) [22]. It was found that low income, low educational level, female gender, and self-reported race as Black or mixed were the main individual factors associated with lower utilization of oral health services, adjusted by the number of decayed teeth and oral health treatment needs. Similar to the present study, some studies developed in Brazil also used data from the PNAD surveys to assess inequalities in oral health care access and found trends in the reduction of inequalities between 2003 and 2008 [23, 24]. These studies also have indicated that there are other aspects that increase the vulnerability of the poorest people. For example, social status is a core factor related to access to social resources, such as formal education and employment, lower access to these resources could lead to a higher exposure to health risk factors [17].

An analysis of the age groups showed that the percentage of non-access, regardless of the year, was higher in children, then decreased in adolescents and adults, and rose slightly in the elderly. It is plausible to hypothesize that there is an expected trend of reduction in the outcome as age increases because a longer lifetime increases the probability of having access to at least one dentist appointment in one’s lifetime. However, we did not observe this trend in the elderly. Although this result seems odd, it is important to highlight that this is a cross-sectional survey study, not a cohort study, which means that the elderly belong to a generation that had fewer opportunities for access to oral health care.

In any case, there was an increase in access to oral health services from 1998 to 2013. From the perspective of plausibility, it is important to highlight that there was an increase in the offer of public oral health services promoted by the PNSB during this period. As mentioned above, the main strategy of this policy was to expand access to public dental services for the whole country through the expansion of oral health teams (ESB) in primary care through the implementation of the Centers of Specialized Dentistry (CEO) in secondary care.

However, reducing the outcome does not guarantee a reduction in health inequalities. Although there has been general improvement in the population’s access to dental services over the years, this improvement may have occurred more slowly for less privileged groups. Further, there may even have been a worsening of health conditions in this population. In some contexts, health policies contribute to the improvement of health indicators without reducing inequalities and, sometimes such improvements lead to the persistence of inequalities or even their expansion [17].

In children, high levels of absolute inequality were maintained, although there was a more pronounced reduction trend compared to other age groups, and relative inequality remained unchanged. This demonstrates that, although the policy promoted an increase in access to dental services for this age group, it may not be as effective for mitigating health inequalities in terms of promoting access for less privileged groups (Table 3).

In adolescents, the absolute inequality (SII) declined from 31.6 in 1998 to 18.2 in 2008 with a slight increase to 19.17 in 2013. The relative inequality was higher in adolescents than in other age groups and similar levels were maintained during this period. The RII found in 2013 was 26.2, which means that among the poorest adolescents, the lack of access to dental care was 26.2 times higher than among the wealthiest adolescents (Table 3).

In adults, there was a lower absolute inequality among all other age groups. Although the reduction in absolute inequality was low, a more pronounced reduction was observed in relation to the relative inequality in comparison to the other age groups. In 2013, the prevalence of the outcome in adults was 18.4 times higher for the poorest (Table 3).

Finally, among the elderly, there was a trend toward decreasing absolute inequality between 1998 and 2008, returning to growth in 2013, where the poorest elderly people presented with a 12.4% greater lack of access than the richest elderly. There was a more pronounced reduction in relative inequality between 1998 and 2003 with similar values in other years. In general, the reduction in inequality was lower among the elderly, which could mean a minor impact of the expansion of access to dental services for this age group (Table 3).

An effective health policy should promote improvements in general health conditions for the population while achieving absolute and relative reductions in inequalities [17]. In the current study, we observed a limitation in the effect of the PNSB, such that, although it increased access to oral health services and consequently decreased the percentage of individuals who never had access to a dentist appointment, there is still room for improvement in overcoming existing income-related inequalities.

It is essential to recognize that contextual and individual characteristics influence the use of dental services. Some characteristics predispose people to use or not use such services, although these conditions are not directly responsible for utilization. Some characteristics are enabling facilitating the use of services, while other characteristics represent the need for utilization, which can be recognized by both the population and health professionals [25]. The implementation of the National Oral Health Policy, evaluated in this study, directly influenced enabling conditions, which facilitated in the financing and organization of services, while for other conditions, this policy had less influence.

In 2020, Brazil has 573 dental schools in operation [26]. Brazilian dentists represent about 12% of all professionals in the world [27], which shows that inequalities in the use of dental services are not related to the lack of suppliers. Moreover, it is relevant to consider that Brazil has a large number of dentists who work in the private sector, with assistance through direct payment and health insurance. In 2014, only 49.9% of Brazilian dentists worked in the public sector and were able to work in the private sector in the same period [28]. Thus, the use of dental services during the period analyzed in this study and hence the inequalities in utilization are also modulated by this market dynamic.

Two important aspects must be considered in relation to these findings. First, the PNSB’s main objective was to promote an increase in the access to oral health services, and there was no specific strategy for reducing inequalities in care in the official documents. Second, there is a limited potential for a specific health policy to impact the comprehensive determinants of inequality. This requires strategies implemented by intersectoral policies that contribute to tackling the problem.

Therefore, the effect of public policies on the trend of inequality must also consider the evolution of the social indicators in Brazil. In addition to the increase in the minimum wage in this period, there was a reduction in income inequality, a significant decrease in poverty and food insecurity, and an increase in access to public services by the poorest and most vulnerable population, resulting in the improvement of several aspects of living conditions of the Brazilians population. Are observed a systematic reduction in income disparities indicator in the analyzed period, the highest since the beginning of its measurement in 1960. The evolution of these indicators, especially after 2003, cannot be credited only to economic factors such as the decrease in unemployment and an increase in the minimum wage. Mainly due to a deliberate effort by public policies such as “Brazil without poverty” (Brasil sem Miséria in Portuguese) and also cash transfer programs such as the Bolsa Família Program and the Continuous Benefit. Nevertheless, the socioeconomic gradient in a given year will always be an expression of inequality [29].

Beyond the need for reformulation to achieve better results, especially regarding the reduction of inequalities in access, the PNSB is not a state policy yet, and has suffered threats due to fiscal adjustment policies and progressive cuts in health resources. Additionally, several factors influenced the perceived importance of the PNSB at the federal level, aggravated by the minor importance of this policy gave by the government, which competes with supposed more relevant health policies [5].

One of the main features of the PNSB is the removal of barriers regarding the ability to pay for the use of dental consultations. The dental service is free of cost at the time of attendance, which differs from the oral health policies developed in some other countries. However, other barriers to access might impede the free utilization, such as geographical barriers, the larger demand for care, insufficient coverage of Family Health Teams in all territories, and the lack of both structure and supplies for dental assistance.

This study has strengths, such as its comprehensive feature, presenting a representative sample of the country during the period assessed. Furthermore, the sample was the result of national household-based surveys with a reliable sample design. Regarding data collection, methodological rigor and the use of validated and comparable instruments must be emphasized, which supports reliability of the data. The standardization of income strata based on the minimum wage in each year allowed comparability in the assessment of inequality. Moreover, the relevance of the assessment of socioeconomic inequalities in access to dental services must be highlighted during a period that is politically important for oral health care in Brazil.

Some limitations can be noted for this study. First, there may have been potential memory bias related to health services utilization, although this kind of bias is a core characteristic of any cross-sectional study using self-reported data. Another limitation is that we were unable to include other factors about oral health inequality due to their unavailability in these Brazilian datasets.

## Conclusions

The findings demonstrate that there was a reduction in the percentage of individuals who never had access to a dentist appointment, as well as a reduction in the absolute inequality of this outcome 10 years after the implementation of the National Oral Health Policy. On the other hand, despite the policy implementation, the maintenance of high levels of relative inequality in access to dental consultation was observed.

Thus, it can be hypothesized that the National Oral Health Policy was very important for expanding public and free of charge access to dental consultation. However, at this stage of policy implementation, it is necessary to assess how policy implementation has contributed to the maintenance of high levels of inequality in access to dental consultation and which strategies are needed to overcome this challenge.

Thus, an important point for debate is the mitigating potential of health inequalities for universal measures focused on the expansion of access to dental care. This argument is based on the assumption that differences in access to dental services are unacceptable. Even though inequalities are mediated by economic position, they should not persist in a country that offers free public dental care with universal coverage. Health inequalities must be included in the political agenda, so that they are foreseen in the construction of more comprehensive policies for addressing the wider determinants of inequality.

## Data Availability

The datasets supporting the conclusions of this article are available on the website of the Brazilian Institute of Geography and Statistics [https://www.ibge.gov.br/estatisticas/downloads-estatisticas.html].

## Abbreviations

CEO: Centers of Specialized Dentistry
DMF-T: Decayed, missing, and filled teeth index
ESB: Oral health teams in primary care
IBGE: Brazilian Institute of Geography and Statistics
PNAD: National Household Sample Survey
PNS: Brazilian National Health Survey
PNSB: National Oral Health Policy
PSU: Primary sample unit
RII: Relative index of inequality
SBBrasil Project 2010: National Oral Health Survey
SII: Slope index of inequality
